# Frailty index transitions over eight years were frequent in The Irish Longitudinal Study on Ageing

**DOI:** 10.12688/hrbopenres.13286.1

**Published:** 2021-06-09

**Authors:** Roman Romero-Ortuno, Peter Hartley, Silvin P. Knight, Rose Anne Kenny, Aisling M. O’Halloran

**Affiliations:** 1 The Irish Longitudinal Study on Ageing, Trinity College Dublin, Dublin, Ireland; 2 Discipline of Medical Gerontology, School of Medicine, Trinity College Dublin, Dublin, Ireland; 3 Mercer’s Institute for Successful Ageing, St James’s Hospital, Dublin, Ireland; 4 Department of Public Health and Primary Care, University of Cambridge, Cambridge, UK

**Keywords:** Aged, Frailty, Longitudinal, Surveys, Transition, Multi-state

## Abstract

**Background**: The frailty index (FI) is based on accumulation of health deficits. FI cut-offs define non-frail, prefrail and frail states. We described transitions of FI states in The Irish Longitudinal Study on Ageing (TILDA).

**Methods**: Participants aged ≥50 years with information for a 31-deficit FI at wave 1 (2010) were followed-up over four waves (2012, 2014, 2016, 2018). Transitions were visualized with alluvial plots and probabilities estimated with multi-state Markov models, investigating the effects of age, sex and education.

**Results**: 8174 wave 1 participants were included (3744 men and 4430 women; mean age 63.8 years). Probabilities from non-frail to prefrail, and non-frail to frail were 18% and 2%, respectively. Prefrail had a 19% probability of reversal to non-frail, and a 15% risk of progression to frail. Frail had a 21% probability of reversal to prefrail and 14% risk of death. Being older and female increased the risk of adverse FI state transitions, but being female reduced the risk of transition from frail to death. Higher level of education was associated with improvement from prefrail to non-frail.

**Conclusions**: FI states are characterized by dynamic longitudinal transitions and frequent improvement. Opportunities exist for reducing the probability of adverse transitions.

## Companion paper

This article is based on methodology first reported in Roman Romero-Ortuno, Peter Hartley, James Davis, Silvin P. Knight, Rossella Rizzo, Belinda Hernández, Rose Anne Kenny, Aisling M. O'Halloran, *Transitions in frailty phenotype states and components over 8 years: Evidence from The Irish Longitudinal Study on Ageing*, Archives of Gerontology and Geriatrics, Volume 95, 2021, https://doi.org/10.1016/j.archger.2021.104401 ^1^.

## What is new?

•    We described longitudinal transitions in frailty index states.

•    Transition probabilities were estimated with multi-state models.

•    Frail had a 21% probability of reversal to prefrail and 14% risk of death.

•    Frailty index transitions are dynamic and include improvement.

•    Opportunities exist for reducing the probability of adverse transitions.

## Introduction

As populations get older, the association between chronological age and health status becomes increasingly variable, to the extent that for a large sector of the older population, chronological age is not a relevant marker for understanding the experience of ageing ^2^. To describe this heterogeneity in health status as we age, the concept of frailty has been proposed ^3– 5^.

The frailty index (FI) methodology was introduced by Rockwood and colleagues ^6, 7^ as a way to quantify the accumulation of people’s health ‘deficits’ (i.e. symptoms, clinical signs, medical conditions and disabilities) at a given chronological age. As per published standard procedure ^8^, a FI can be constructed on any suitable health database by considering a minimum of 30 deficits that need to satisfy the following criteria: (a) be associated with health status, and not simply attributes (e.g. hair graying); (b) cover a range of systems; (c) not saturate too early (e.g. presbyopia is nearly universal by age 55); and (d) their prevalence must increase with age (excluding survivor effects); in addition, in repeated assessments the FI construction must be the same ^8^.

Since FI deficits must increase with age, the FI has a statistically significant association with chronological age ^9^. However, on account of the above-mentioned population heterogeneity, the effect size of this association has been found to be small ^10, 11^. The sex-specific properties of the FI have also been studied. A systematic review and meta-analysis ^12^ consistently showed that women have higher FI scores than males at all ages. However, whilst women tend to accumulate more deficits than men of the same age, their risk of mortality tends to be lower ^6^. Socioeconomic status, including education, has also been reported to explain variation in FI within individuals of the same chronological age ^13^. 

Frailty in older adults can be improved and even reversed with appropriate medical and non-medical interventions ^14^. However, despite abundant research to the contrary, non-specialist clinicians and the general public often believe that frailty is a ‘fixed’ state with little potential to change over time ^15^. Previous works have shown that the FI is longitudinally dynamic ^16– 21^, but Irish data on FI transitions was lacking and few studies have employed long follow-up periods. Our aim was to describe the eight-year longitudinal transitions of FI states using data from The Irish Longitudinal Study on Ageing.

## Methods

### Design and setting

We analyzed data from a population-based longitudinal study that collects information on the health, economic and social circumstances from people aged 50 and over in Ireland (The Irish Longitudinal Study on Ageing: TILDA). Wave 1 of the study (baseline) took place between October 2009 and February 2011, and subsequent data was collected approximately two-yearly over four longitudinal waves (wave 2: February 2012 to March 2013; wave 3: March 2014 to October 2015; wave 4: January to December 2016; wave 5: January to December 2018). An overview of the study is available on https://tilda.tcd.ie/about/where-are-we-now/. The full cohort profile has been described elsewhere ^22, 23^.

### Sample

 The baseline analytical sample included participants who had complete FI information at Wave 1. For subsequent waves, information was collected on transitions in FI states and attrition due to deaths or missing data. 

### Construction of the FI

As previously published ^24^, a 31-item FI was constructed using self-reported health measures available in TILDA’s Computer-Assisted Personal Interview (CAPI) questionnaire conducted at wave 1. The selection of deficits was consistent with the standard FI requirements ^8^, including that deficits are any symptom, sign, disease or disability associated with age and adverse outcomes, are present in at least 1% of the population, cover several organ systems, and have under 5% missing data ^24^. The components of this 31-item FI are in Appendix 1 (see *Extended data*) ^25^. Deficits with more than two categories (i.e. no=0 or yes=1) were coded as a proportion of the number and order of responses; for example, five-answer categories for the deficit ‘Self-rated physical health’: *Excellent*, *Very good* and *Good* were coded as 0 (no deficit); *Fair* was coded as 0.5 (partial deficit); and *Poor* was coded as 1.0 (full deficit). Analyses from diverse datasets have suggested that variables included in an FI can be coded either as dichotomous or ordinal, with negligible impact on the performance of the index in predicting mortality ^26^.

In keeping with previous literature ^27^, the following cut-offs were applied at each wave for the definition of the three FI states: FI < 0.10: non-frail; FI ≥ 0.25: frail; and the rest: prefrail. As conducted by others ^28^, and as a sensitivity analysis, we categorized the FI based on baseline quartiles.

### Other measures

Age was measured at baseline and each wave, and the following were measured at baseline: sex (male = 0; female = 1); and highest education level (primary or less = 1; secondary = 2; third/higher = 3).

### Mortality

Mortality was ascertained for all study participants at each follow-up wave. TILDA has approval from Ireland’s Central Statistics Office to link survey respondents to their death certificate information held centrally by the General Register Office, where every death in the Republic of Ireland must be registered ^29^. Other than deaths, attrition at each wave was classified as ‘missing’.

### Statistical analyses

Descriptive statistics were computed with IBM SPSS Statistics version 25 (IBM Corp., Armonk, NY, USA) and given as mean with standard deviation (SD) and range or proportion (%).

For the visualization of the longitudinal trajectories of FI states, an alluvial chart was created using the R ggalluvial package ^30^. In the alluvial plot, the height of the stacked bars at each wave (which represent whether participants’ status for the given frailty state was yes, no, missing or died) is proportional to the number of participants identified as belonging to this state at each wave. The thickness of the streams connecting the stacked bars between waves are proportional to the number of participants who have the state identified by both ends of the stream. As a supplementary visualization, alluvial charts were created for two age subsamples: less than 75 and 75 or more at baseline. As a further supplementary visualization, alluvial charts were created for each of the individual 31 FI items on the total sample.

To estimate transition probabilities for the FI states, we used multi-state Markov models using the R msm package, which allows a general multi-state model to be fitted to longitudinal data ^31^. The multi-state Markov model is a way of describing a process in which individuals move through a series of states over time. All missing data were censored and considered missing completely at random. In addition, we conducted sensitivity analyses where missing data was modelled as an additional state in the models. We obtained matrices of estimated transition probabilities from wave x to wave x + 1 (with 95% confidence intervals [CIs]) for each FI state. We adjusted the multi-state models for age, sex and education. Multi-state models handle confounders at baseline and subsequent waves. Whilst sex and education remained constant across waves, the age covariate was time-varying (i.e. increased for each wave). Hazard ratios (HRs) and 95% CIs for the estimated covariate effects of age, sex and education were obtained. HRs were considered significant when their CIs did not include 1.

### Ethics

Ethical approval for each wave was obtained from the Faculty of Health Sciences Research Ethics Committee at Trinity College Dublin, Ireland: Wave 1: "The Irish Longitudinal Study on Ageing (granted 2 May 2008)"; Wave 2: "The Irish Longitudinal Study on Ageing (granted 19 October 2011)"; Wave 3: “Main Wave 3 Tilda Study (granted 9 June 2014)”; Wave 4: "Ref: 150506"; and Wave 5: "Ref: 170304". Prior to inclusion in the study, all participants provided written informed consent for participation and utilisation of collected data for scientific publications.

## Results

TILDA wave 1 recruited a total of 8504 participants, of whom 330 (3.9%) were aged less than 50 years. The remaining 8174 had complete FI information (3744 men and 4430 women). The mean (SD; minimum, maximum) age of wave 1 participants (n=8174) was 63.8 (9.8; 50–105) years; for wave 2 (n=6994): 65.5 (9.5; 52–97); for wave 3 (n=6249): 67.5 (9.2; 54–98); for wave 4 (n=5571): 69.2 (8.9; 56–101); and for wave 5 (n=4874): 70.6 (8.5; 58–103). Overall, 6832 (83.6%) participants were aged <75 and 1342 (16.4%) 75 or more. The counts and proportions for FI states and deaths at each wave is presented in Table 1.

**Table 1.** Proportions of frailty index states and deaths at each wave.

	Wave 1	Wave 2	Wave 3	Wave 4	Wave 5
Non-frail	55.5% (n=4540)	52.5% (n=3667)	50.9% (n=3179)	50.1% (n=2794)	46.6% (n=2273)
Prefrail	30.7% (n=2508)	32.4% (n=2262)	34.3% (n=2140)	35.0% (n=1952)	36.7% (n=1790)
Frail	13.8% (n=1126)	15.2% (n=1061)	14.9%(n=928)	14.8%(n=826)	16.6%(n=810)
Deaths	0.0% (n=0)	2.5% (n=208)	3.8% (n=309)	3.2% (n=259)	3.4% (n=275)

**Figure 1.**
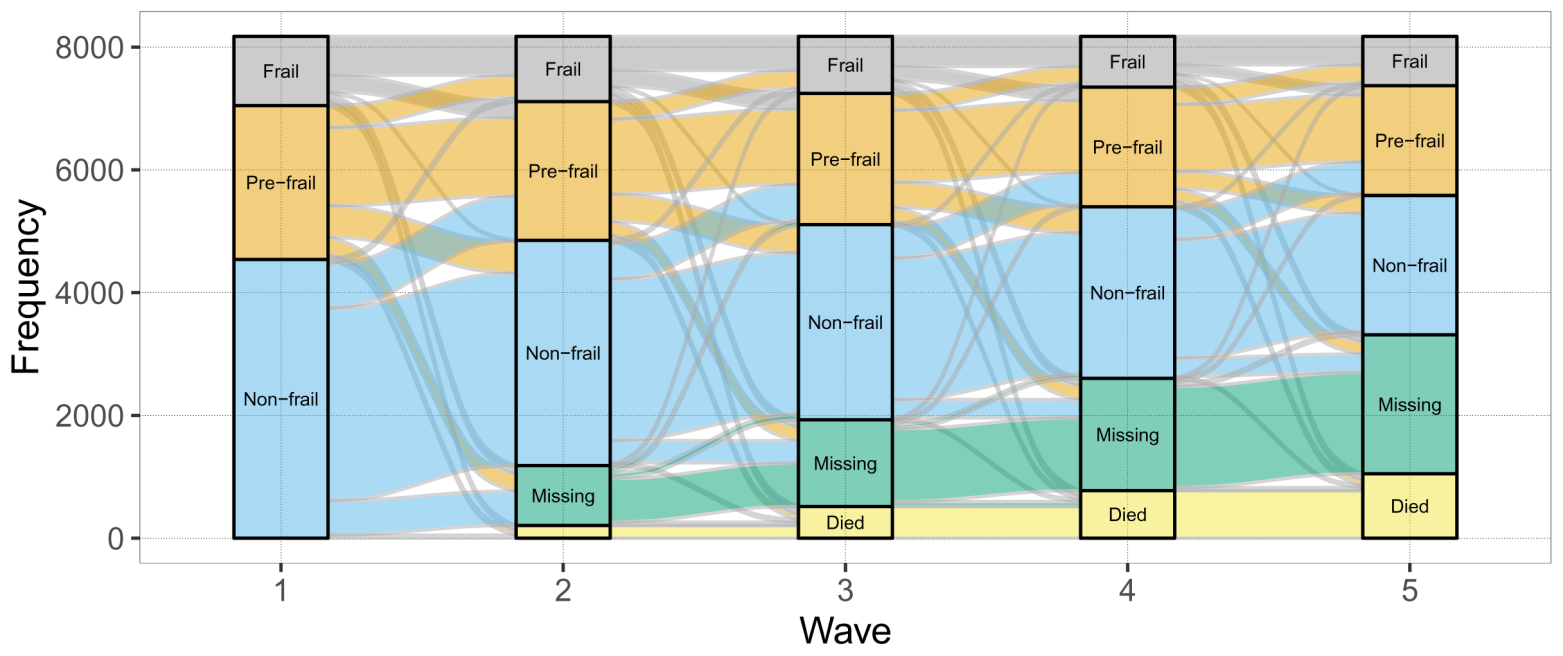
Alluvial chart of the longitudinal transitions of frailty index states in The Irish Longitudinal Study on Ageing (n=8174).

The alluvial plot for the FI states in the total sample is shown in Figure 1, and Appendices 2 and 3 (see *Extended data*) show the alluvial plots for age groups (<75 versus 75 and more), and each of the 31 FI items in the total sample, respectively. As expected, the cumulative proportions of deaths and missing data increased across waves. Numbers of FI state transitions in the total sample are detailed in Appendix 4 (see *Extended data*) ^25^.

**Figure 2.**
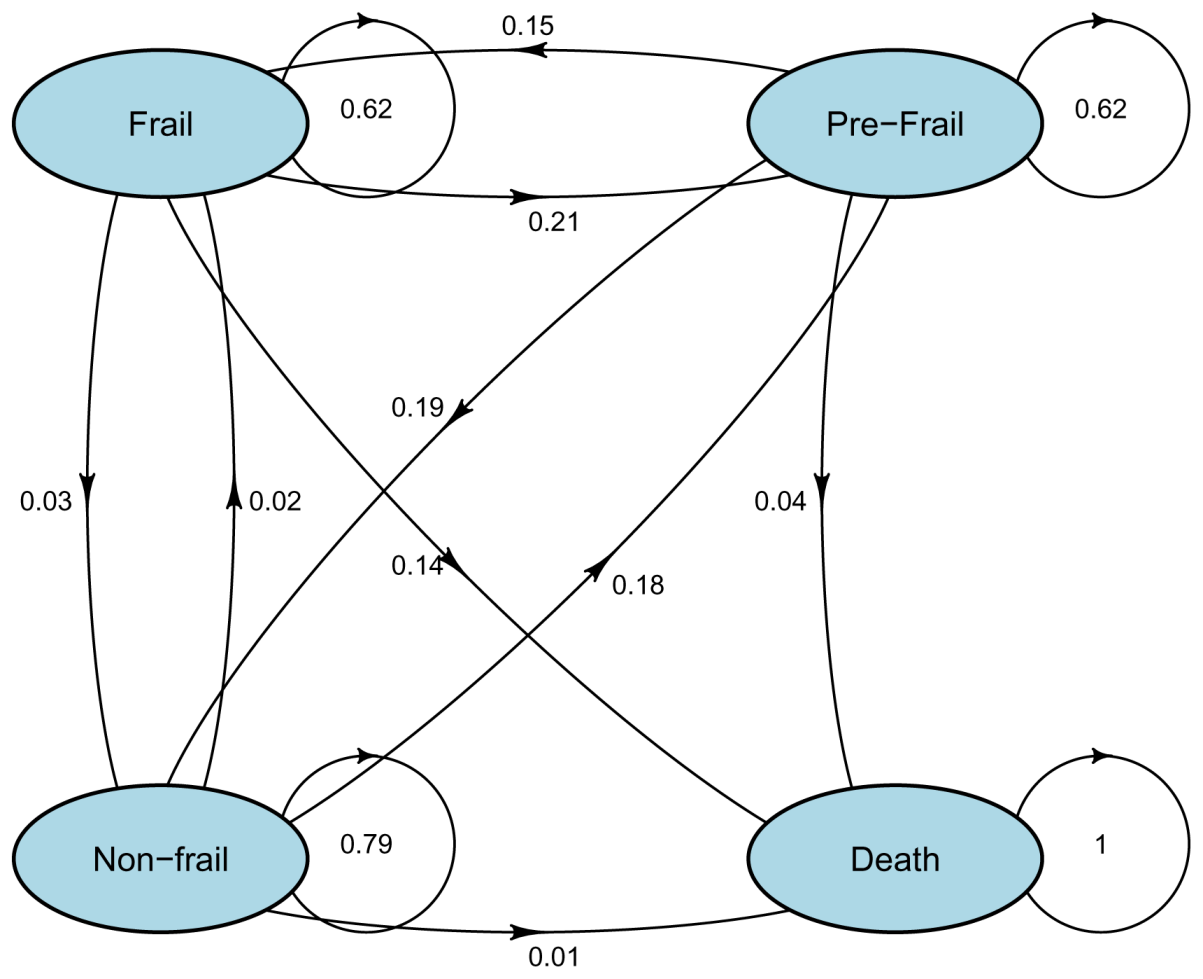
Estimated transition probability for each frailty index state (from wave x to wave x + 1) in the total sample (n=8174).

Table 2 shows the probabilities of transition (with 95% confidence intervals) in frailty states from one wave to the next. Figure 2 visually shows the transition probabilities. In the age subanalyses presented in Appendix 5 (see *Extended data*) ^25^, in those age 75 or more, the risk of death from a frail state was 67%, and the probabilities of improvements from frail to prefrail and prefrail to non-frail were 12% and 6%, respectively.

**Table 2.** Estimated transition probability (and 95% CI) matrix for each frailty index state (from wave x to wave x + 1) in the total sample (n=8174).

	STATE TO			
STATE FROM	Non-frail	Prefrail	Frail	Death
**Non-frail**	0.79 (0.78, 0.79)	0.18 (0.17, 0.19)	0.02 (0.02, 0.02)	0.01 (0.01, 0.01)
**Prefrail**	0.19 (0.18, 0.20)	0.62 (0.60, 0.63)	0.15 (0.15, 0.16)	0.04 (0.03, 0.05)
**Frail**	0.03 (0.03, 0.04)	0.21 (0.20, 0.22)	0.62 (0.60, 0.63)	0.14 (0.13, 0.16)

CI: confidence interval.

Appendix 6 ( *Extended data*) ^25^ shows the transition probabilities based on FI quartiles at baseline in the total sample. According to this FI categorization, severe frailty had a 25% risk of death, a 22% probability of transition to moderate frailty and a 6% probability of improvement to mild frailty. The probability of improvement from moderate to mild frailty was 26%, and the probability of improvement from mild frailty to fit state was 22%. Other transition probabilities according to the FI quartiles categorization are shown in Appendix 6 (see *Extended data*) ^25^.

Appendix 7 ( *Extended data*) ^25^ shows a reanalysis modelling missing data as a fourth state. Table 3 shows the effects of sex, age and education in the multi-state models. Being older increased the risk of adverse state transitions from frail to death, from prefrail to frail, and from non-frail to prefrail. The opposite was suggested for favourable transitions from frail to prefrail, and prefrail to non-frail.

**Table 3.** Hazard ratios and 95% CIs of the estimated covariate effects of sex, age and education in the multi-state models.

From - To	Sex = Female	Age	Education = Secondary	Education = Third/Higher
**Frail - Prefrail**	**0.83 (0.72, 0.95)**	**0.97 (0.97, 0.98)**	1.00 (0.86, 1.17)	1.19 (1.00, 1.42)
**Frail - Non-frail**	0.79 (0.00, 1259.15)	0.94 (0.62, 1.42)	1.01 (0.00, 4101.10)	1.51 (0.00, 19123.09)
**Frail - Death**	**0.64 (0.44, 0.93)**	**1.14 (1.11, 1.17)**	0.98 (0.62, 1.56)	1.55 (0.96, 2.52)
**Prefrail - Frail**	**1.32 (1.17, 1.49)**	**1.04 (1.04, 1.05)**	**0.69 (0.60, 0.79)**	**0.61 (0.53, 0.71)**
**Prefrail - Non-frail**	**0.84 (0.76, 0.93)**	**0.97 (0.96, 0.98)**	1.04 (0.91, 1.18)	**1.23 (1.08, 1.40)**
**Prefrail - Death**	0.60 (0.27, 1.36)	1.06 (1.01, 1.11)	1.31 (0.39, 4.40)	0.39 (0.05, 2.96)
**Non-frail - Frail**	1.73 (0.07, 45.67)	0.79 (0.61, 1.01)	0.89 (0.00, 950.30)	0.19 (0.00, 270.39)
**Non-frail - Prefrail**	**1.22 (1.12, 1.32)**	**1.05 (1.05, 1.06)**	**0.76 (0.68, 0.85)**	**0.68 (0.60, 0.76)**
**Non-frail - Death**	0.32 (0.01, 6.93)	1.13 (0.99, 1.29)	0.44 (0.00, 119.96)	1.37 (0.03, 65.28)

CI: confidence interval. Significant associations (where the CI does not include 1.00) are depicted in bold.

As regards sex, being female increased the risk of adverse transitions from non-frail to prefrail, and prefrail to frail; however, it reduced the risk of transition from frail to death. Being female reduced the risk of favourable transitions from pre-frail to non-frail and frail to prefrail. In terms of education, there were trends in the expected direction with higher levels of education being positively associated with the favourable transition from prefrail to non-frail and negatively associated to adverse transitions ( Table 3).

## Discussion

Using Irish data from a large population-based study of ageing spanning eight years, we corroborated that FI states are dynamic and many transitions are affected by age, sex, and education, in the expected directions. Indeed, frailty is not all steady state and progression, but reversion is also common ^32^. Our study adds value to previous research by reporting a long follow-up period in an Irish sample and offers some new insights on the dynamics of the FI in relation to chronological age. Indeed, our age subanalyses suggested that the FI dynamics are not the same in older groups, with frailer people aged 75 or more having higher mortality and less reversibility than people aged less than 75. This agrees with previous research suggesting that chronological age and the FI may be complementary in predicting health outcomes ^33, 34^. Specifically about sex, our results are in keeping with the known fact that whilst women tend to accumulate more deficits than men of the same age, their risk of mortality tends to be lower ^6^. Our results also agree with previous observations that sociodemographic factors (e.g. education) are related to changes in FI status ^16^. The age-sex-education effects are consistent with previous research and we did not model other time-varying covariates such as physical activity or polypharmacy ^17^. However, in our FI operationalization, items related to physical activity difficulties and polypharmacy were included as defining FI deficits (Appendix 1, *Extended data*) ^25^. On the other hand, the efficient statistical handling of additional covariates would have probably required a larger sample size, judging by some of the wide CIs obtained in Table 3 for transitions with a relatively low number of events (Appendix 4, *Extended data*) ^25^. Even though we broke the FI into three categories utilizing a previously reported scheme and performed sensitivity analysis based on quartiles, the FI is continuous in nature and concern remains as to its optimal categorization ^27^.

Our study has further limitations. For the mortality outcome, specific causes of death were not studied, and addressing this in future studies could shed light into specific biological risks associated with FI states. Another limitation is that missing data was censored as missing completely at random. However, analyses in Appendix 7, *Extended data* ^25^, suggested that frailer individuals were not more likely to have missing data at future waves (11% for all frailty states).

Another limitation of the use of an FI that was based on self-report is measurement error or misclassification. As visually suggested by the individual-deficit alluvial plots in Appendix 3 (see *Extended data*) ^25^, some items showed implausible favourable transitions (i.e. from having history of a medical condition at one wave, to not reporting history of that same medical condition at the following wave). However, Appendix 8 ( *Extended data*) ^25^ shows, for example, that implausible transitions from having to not having history of heart attack, diabetes, osteoporosis, cancer, and stroke/TIA, were less frequent (n = 155 to 657) than transitions from other deficits where improvement could be more plausible (e.g. self-rated health, daytime sleepiness, self-rated memory, and difficulties rising from a chair or carrying weights, n = 1946 – 4060). Research from other longitudinal studies has shown that self-reported health questions are prone to significant biases ^35^, and TILDA is not free of those.

As a limitation to the extrapolation of the study and its external validity, it is important to note that the operationalization of frailty does not have a universal consensus, and we here opted for the FI model. Hence, our results cannot be extrapolated to other frailty models such as the frailty phenotype ^5^. In the latter case, polypharmacy is not included in the definition of frailty; hence, the frailty phenotype may be more suited for the study of that covariate than the FI. However, the frailty phenotype would be less suited for the study of physical activity because that item is included in the frailty definition.

In summary, given the importance of FI states transition information in planning public health interventions, there is a need to support data collection and projects that measure frailty trajectories and transitions between different levels of frailty severity ^36^, in a way that non-specialist clinicians and the general public can easily understand. We believe that it is important to create a body of international evidence that consistently supports the important public health message that frailty is dynamic over a long period of time, throughout which there is potential and opportunities for improvement. In future work, it would be possible to adapt more advanced methodologies ^37, 38^ to explore the main clusters or groupings of factors that determine different trajectories to identify the best opportunities for reducing the probability of adverse frailty transitions.

## Data availability

### Underlying data

The data underlying the results cannot be shared due to ethical and data protection issues. Requests to access this data can be made directly to TILDA ( tilda@tcd.ie) and will be considered on a case-by-case basis. The first four waves of TILDA data are available from the Irish Social Science Data Archive (ISSDA) at www.ucd.ie/issda/data/tilda/. To access the TLDA survey data, please complete an ISSDA Data Request Form for Research Purposes, sign it, and send it to ISSDA by email ( issda@ucd.ie).

### Extended data

Figshare: Extended data.docx. https://doi.org/10.6084/m9.figshare.14681292.v1 ^25^.

This project contains the following extended data:

- Appendix 1 (31-item Frailty Index (FI): items and scoring of individual items)
- Appendix 2 (Alluvial charts by age groups)
- Appendix 3 (Alluvial charts for each of the 31 FI items in the total sample)
- Appendix 4 (Numbers of transitions for FI states in the total sample)
- Appendix 5 (Sensitivity analysis of transition probabilities by age groups)
- Appendix 6 (Sensitivity analysis of transition probabilities on the total sample categorised by FI quartiles at baseline)
- Appendix 7 (Sensitivity analysis where missing data was considered as an additional state in the multi-state models)
- Appendix 8 (Implausible transitions from having to not having history of medical conditions were less frequent than transitions from other deficits where improvement would be more plausible)

Data are available under the terms of the Creative Commons Attribution 4.0 International license (CC-BY 4.0).
