# Supplementary material for: Frailty index transitions over eight years were frequent in The Irish Longitudinal Study on Ageing
Source: HRB Open Res. 2021 Jun 9;4:63. [Version 1] doi: 10.12688/hrbopenres.13286.1 (PMC8406448; doi:10.12688/hrbopenres.13286.1)
Supplement: Peer review history

# Peer review: Reviewer response for version 1

**DOI:** 10.21956/hrbopenres.14461.r30074

**Published:** 2021-09-03

**Authors:** Mario Ulises Pérez Zepeda

**Affiliations:** 1 Instituto Nacional de Geriatría, Mexico City, Mexico

I enjoyed very much reading this article and its companion paper. There is no doubt that that the field of frailty needs to advance into the direction of longitudinal analyses and in particular of the meaning of the trajectories. In this work, the authors show how these transitions and trajectories of frailty, and depicted with the frailty index, change over time and how some factors are associated with this change, mainly sex, age and education. In addition, in their ancillary analyses, show how different arranges bring light into for example age (age stratified analysis) or the dynamics of the deficit accumulation. Methods are described in detail, and authors make their best to address methodological/analytical problems that are common when doing research on frailty index with data sets. I missed that the authors did not discuss the difference/similarity of the phenotype with their results with the FI. They already have one work on this matter with the phenotype, an opinion on how do this two tools compare would be mostly appreciated by the 'frailty community' It is true that the index and the phenotype are different, as authors acknowledge, however, they live under the same semantic umbrella, frailty; and that should not be overlooked. Even if the authors think they are measuring different conditions (for example the phenotype could be a great tool for measuring sarcopenia); would be interesting to read their thoughts on that. As they iteratively comment, there is scarce evidence on this matter, and having first hand the opinion from researchers that had the opportunity to address the phenomenon both with the index and the phenotype will certainly enrich the discussion. Furthermore, what would they think these trajectories will look like with other tools? Maybe this is too far from their objective, but a brief comment might increase interest and raise more interesting questions. Just a technical problem, I did not find the alluvial graphs for each deficit.

Is the work clearly and accurately presented and does it cite the current literature?

Yes

If applicable, is the statistical analysis and its interpretation appropriate?

Yes

Are all the source data underlying the results available to ensure full reproducibility?

Yes

Is the study design appropriate and is the work technically sound?

Yes

Are the conclusions drawn adequately supported by the results?

Partly

Are sufficient details of methods and analysis provided to allow replication by others?

Yes

Reviewer Expertise:

Geriatrics, frailty, geriatric syndromes, geriatric epidemiology, sarcopenia

I confirm that I have read this submission and believe that I have an appropriate level of expertise to confirm that it is of an acceptable scientific standard.

# Peer review: Reviewer response for version 1

**DOI:** 10.21956/hrbopenres.14461.r29644

**Published:** 2021-08-27

**Authors:** Danielle Ni Chroinin

**Affiliations:** 1 South Western Sydney Clinical School, UNSW, Sydney, NSW, Australia; 2 Liverpool Hospital, Sydney, NSW, Australia

Thank you for the opportunity to review this paper, which details transitions in frailty with time over an 8 year period as part of the Irish TILDA project. The study includes adults in later middle age through to old age (50+ years, mean 63.8 years at recruitment; >80% aged <75), with >8000 participants included in Wave 1, and data available for 4874 by wave 5. The authors highlight that a companion paper, which adopted different focus and definitions of frailty, exists and was published elsewhere.

 Overall, the paper is clearly written, and the diagrams and tables present what could be very complicated data in an accessible fashion. Follow-up rates are slightly disappointing, but perhaps not unexpected. The rate of death is low (and as sourced from the central register, shouldn’t be influenced by ‘missing’ data’), but the population is not very old, which may explain that figure. Data regarding activities and interventions which might have been associated with frailty transitions (in either direction) are not included, which is a pity, although the authors note that analysing more covariates was beyond the scope of the paper/sample size. The findings add to the evidence base supporting the concept of frailty as a dynamic condition, over a longer (8-year) follow up period, and in an Irish context. Transitions were affected by age, sex and (early) educational attainment, but these are fixed. For clinicians, I think the real key will be identifying what we can do to influence trajectories.

**Further comments:**

 The Introduction gives a clear explanation of frailty and the FI tool, which will be helpful to the uninitiated reader. The authors note that frailty may be reversible and is dynamic. The lack of Irish data and long follow up are highlighted. It would be helpful to know where existing data hail from, and if certain ethnicities/cultural backgrounds are under- or over-represented in the available data, and if/how the Irish population differ from these.

**Methods:**

 Design and setting were outlined concisely, and a link to further information about the TILDA project was included. Construction of the FI is clearly outlined, and the specific data-points collected are included in Appendix 1.

 The cut-offs applied are perhaps slightly arbitrary and non-age-adjusted, based on those used by the first author in an earlier publication, but this is a reasonably pragmatic approach to use of a continuous scale, and quartiles were analysed in a secondary analysis. The authors helpfully included death data from the General Register Office (rather than other maybe less-accurate data sources). Models were adjusted for age, sex and education, but not SES per se.

**Results:**

 Data were available for 4874 of the original 8174 by the end of the final wave (5), with 3% reported dead at the end of 8 year follow up- low by geriatric standards, but maybe not given the relative youth of the recruitees.

 It might have been nice if distribution of deficits in FI (Appendix 1) were included within the Appendix (e.g. proportions with polypharmacy, IHD, DM, stroke, etc, and 5-point distribution for ‘self-rated memory’ etc).

 The baseline rate of frailty/pre-frailty in the cohort aged <75 is not insubstantial.

 Unsurprisingly, likelihood of death in older frail patients was high, and those with severe frailty were at highest risk. While there were transitions in both directions, those with severe frailty rarely reversed to ‘fit’ or ‘mild[ly] frail’ levels, and women more often experienced adverse transitions.

 The alluvial plots are helpful for visual representation, the tables are easy to understand, and the Appendices give additional detail.

**Discussion:**

 While the opening line is perhaps a little underwhelming in stating that the authors ‘corroborate that FI states are dynamic’, agree better this than over-interpretation.

 While the authors acknowledge that they did not ‘model other time-varying covariates such as physical activity or polypharmacy’, changes in activity, geriatric/multidisciplinary team intervention, changes in polypharmacy/medication burden and acute hospitalisations would have been helpful to have known, and I believe that the TILDA dataset would include some of this info (e.g. in the companion paper, info regarding physical activity is captured using the IPAQ-SF). While the authors note that ‘statistical handling of additional covariates would have probably required a larger sample size’, some indication of how these might have related to changes in frailty trajectory would be of interest, especially to clinicians. This is noted in the paper’s concluding line, but the authors have quite a wealth of information within TILDA, and may be able to explore this further in their own dataset.

 A small point: In relation to implausible transitions, I wonder if cognitive impairment might have accounted for some of these. While they were not common, I was unclear as to whether the authors performed additional sensitivity analyses where ‘implausible transitions’ were reanalysed with the original status carried through (e.g. positive ‘history stroke’ in wave 1 extended to all subsequent waves, even if the patient changed their answer to negative in subsequent waves).’

Is the work clearly and accurately presented and does it cite the current literature?

Yes

If applicable, is the statistical analysis and its interpretation appropriate?

Yes

Are all the source data underlying the results available to ensure full reproducibility?

No

Is the study design appropriate and is the work technically sound?

Yes

Are the conclusions drawn adequately supported by the results?

Yes

Are sufficient details of methods and analysis provided to allow replication by others?

Yes

Reviewer Expertise:

Geriatric medicine

I confirm that I have read this submission and believe that I have an appropriate level of expertise to confirm that it is of an acceptable scientific standard.

# Peer review: Reviewer response for version 1

**DOI:** 10.21956/hrbopenres.14461.r29817

**Published:** 2021-08-02

**Authors:** Dorly J. H. Deeg

**Affiliations:** 1 Amsterdam Public Health Research Institute, Epidemiology and Data Science, Amsterdam UMC, Vrije Universiteit Amsterdam, Amsterdam, The Netherlands

This is a companion paper to ‘Transitions in frailty phenotype status and components of frailty over 8 years’ (2021), as stated in the preliminaries. The difference is that a different definition of frailty is now used, i.e., the Frailty Index (FI), which is conceived of as a continuous index, rather than an index with two or three states as is the case with the frailty phenotype (FP). In the current paper, socio-demographic correlates of transitions are assessed. In the companion paper, an emphasis was on transitions in the five components of the FP. As the FI is conceived of as a continuous measure, the wisdom of distinguishing states (and transitions between them) might need some argumentation.

 The argument leading up to the aim of the paper is rather technically phrased: Irish data on FI transitions are not available yet, and few studies have employed longer follow-ups. However, why would additional (i.e., Irish) data on transitions be useful? And why would a longer follow-up be useful? I would have expected a more substantial argument for a study of transitions. The only statement that comes close to an argument is that ‘non-specialist clinicians and the general public often believe that frailty is a “fixed state”’. But the authors also state that there is ‘abundant research to the contrary’, so what does the current study add to the existing research findings? The bullets listed under ‘What is new?’ are not really ‘new’. I know that as a reviewer, I am not asked to comment on novelty or interest of the paper, but a proper argumentation is indispensable.

 I do see merits in this study. It includes a large number of participants, which allows the study of less-frequent transitions. The relatively large number of time intervals across which the transitions are observed, also helps the study of less frequent transitions. That said, for some calculations, the number of transitions still is too low, as apparent from the huge confidence intervals for the association of secondary and higher education with the transition frail-nonfrail and nonfrail-frail (Table 3). Therefore, I would recommend to dichotomise, instead of trichotomise, education.

 Apparently, the main interest of the authors is in back-transitions (‘favourable’ transitions), as they tend to report the risk of death and the probability of improvement, omitting report of risks of ‘adverse’ transitions. The average age of the initial sample is rather young at 64 years; only 16% are older than 74. From Appendix 5, one can observe that the frailty states are much less stable at ages 75+ than at younger ages; in particular, the probability of adverse transitions is much higher, especially to the state of death. As in clinical practice, the majority of frailty cases have ages 75+, the findings in this age group are especially useful. When reporting the results for the full sample, however, the emphasis on favourable transitions suggests that these apply to all ages. This suggestion should be avoided, for example by showing Appendix 5 in the main text and distinguishing older ages in the discussion of the results.

 The cut-point between ‘fit’ and ‘pre-frail’ is chosen as 0.10, referring to a previous publication by the same author(s) (reference 27). However, this article proposes to use age-specific cut-points. Therefore, this article does not support the current cut-off. Other studies use as a criterion for ‘fit’ having a maximum of 4-5 deficits, or – as the authors state – use quartiles. An argument to support the cut-point of 0.10 is needed.

 The alluvial chart nicely illustrates the various transitions, including transitions to missing. Incidentally, it would be more logical to have the categories ‘dead’ and ‘missing’ stacked at the top rather than at the bottom. From Appendix 7, it can be observed that there are transitions from missing back to having available data or death. Regardless, missing is the most stable state at probability 0.82. Are they all alive at the end of the study? In their Discussion, the authors mention that all frailty states had similar transition probabilities to ‘missing’. How does this reflect on their assumption of MCAR?

 Because frailty is, by definition, an unstable state, many changes can happen within the 2-year interval between waves. This should be commented on in the Discussion, including the consequences for the transition probabilities found. Are they under- or overestimated?

 Table 2 should include the absolute numbers, as now provided in Appendix 4 only – which then can be omitted. The absolute numbers should be available in the main text. The same goes for Appendix 5. Please also state the total number of transitions, not only the initial number of participants. The three states presented in this table appear highly stable, with probabilities of staying in the same state as 0.79, 0.62, and 0.62, respectively. How is it possible that in Appendix 5, the age-specific probabilities of staying in the same state are so much lower?

 The authors mention in the Discussion that 5 of the 31 items, all self-reported chronic diseases, show implausible favourable transitions. This ‘recovery’ should indeed be considered as a measurement error, which can be corrected by proper longitudinal cleaning of the data. I recommend that the authors clean their data longitudinally and then recalculate the FI transition probabilities.

**Details:**

- In Table 1, the last row should read ‘Deaths since previous wave’.
- Appendix 5 is not a ‘sensitivity analysis’ as stated in its header, but a sub-analysis, as stated in the main text.
- In the very last sentence of the Discussion, it would be good to insert ‘frailty’ before ‘trajectory’.

Is the work clearly and accurately presented and does it cite the current literature?

Partly

If applicable, is the statistical analysis and its interpretation appropriate?

Yes

Are all the source data underlying the results available to ensure full reproducibility?

Yes

Is the study design appropriate and is the work technically sound?

Partly

Are the conclusions drawn adequately supported by the results?

Partly

Are sufficient details of methods and analysis provided to allow replication by others?

Yes

Reviewer Expertise:

Epidemiology of ageing; longitudinal studies

I confirm that I have read this submission and believe that I have an appropriate level of expertise to confirm that it is of an acceptable scientific standard, however I have significant reservations, as outlined above.

# Peer review: Reviewer response for version 1

**DOI:** 10.21956/hrbopenres.14461.r29766

**Published:** 2021-07-21

**Authors:** Daniel Kam Yin Chan

**Affiliations:** 1 School of Public Health and Community Medicine, UNSW, Sydney, NSW, Australia

The study is well designed and the paper is well written. An excellent study.

 The only query (not criticism) is whether intervention data (medical or non-medical) are being collected, and if yes, are they available? If not available, it would be good to indicate it in the paper, such as under the section "other measures", as I cannot find it there. This would be of interest to indicate if such interventions improve the reversibility in different stages of Frailty Index.

Is the work clearly and accurately presented and does it cite the current literature?

Yes

If applicable, is the statistical analysis and its interpretation appropriate?

Yes

Are all the source data underlying the results available to ensure full reproducibility?

Yes

Is the study design appropriate and is the work technically sound?

Yes

Are the conclusions drawn adequately supported by the results?

Yes

Are sufficient details of methods and analysis provided to allow replication by others?

Yes

Reviewer Expertise:

Geriatrics

I confirm that I have read this submission and believe that I have an appropriate level of expertise to confirm that it is of an acceptable scientific standard.
